# Role of intestinal extracellular matrix-related signaling in porcine epidemic diarrhea virus infection

**DOI:** 10.1080/21505594.2021.1972202

**Published:** 2021-09-13

**Authors:** Yuchen Li, Jianda Li, Xiuyu Wang, Qingxin Wu, Qian Yang

**Affiliations:** Moe Joint International Research Laboratory of Animal Health and Food Safety, College of Veterinary Medicine, Nanjing Agricultural University, Jiangsu, PR China

**Keywords:** Extracellular matrix, porcine epidemic diarrhea virus, cluster of differentiation 44, serpin family E member 1, viral replication, viral release

## Abstract

Porcine epidemic diarrhea virus (PEDV) is emerging as a major threat to the global swine industry. Clinical PEDV infection is associated with severe intestinal lesions, resulting in absorptive dysfunction and high mortality rates in suckling piglets. The extracellular matrix (ECM) is an important component of intestinal tissue, providing a structural framework and conveying tissue-specific signals to nearby enterocytes. In this study, we investigated the extensive ECM remodeling observed in intestinal epithelial cells infected with PEDV and elucidated the associated activated ECM receptor-related pathways. Protein-protein interaction network analysis revealed two significantly differentially expressed genes (cluster of differentiation 44 [*CD44*] and serpin family E member 1 [*SERPINE1*]) associated with the ECM. At the transcriptional level, both genes exhibited significant positive correlation with the extent of PEDV replication. Similarly, the expression of CD44 and PAI-1 (encoded by *SERPINE1*) was also increased in the intestines of piglets during viral infection. Furthermore, CD44 exhibited antiviral activity by enhancing the expression of antiviral cytokines (e.g., interleukin [IL]-6, IL-18, IL-11, and antimicrobial peptide beta-defensin 1) by activating nuclear factor-κB signaling. Conversely, PAI-1 was found to promote the release of progeny virions during PEDV infection, despite a decreased intracellular viral load. Nevertheless, the underlying mechanisms are still unclear. Taken together, our results highlighted the biological roles of specific ECM-regulated genes, i.e., *CD44* and *SERPINE1* in suppressing and promoting PEDV infection, thereby providing a theoretical foundation for the role of the ECM in intestinal infections and identifying potential therapeutic targets for PEDV.

## Introduction

Porcine epidemic diarrhea virus (PEDV), a prominent member of the coronavirus family, has caused major economic losses within the global pig industry [1]. PEDV-infected piglets have been shown to exhibit severe intestinal mucosal injury, characterized by extensive enterocyte necrosis, villous atrophy, and shedding. These symptoms result in increased permeability and absorptive dysfunction of the intestinal tissue, ultimately leading to acute watery diarrhea and death by dehydration in suckling piglets [2]. Thus, the maintenance of intestinal mucosal barrier integrity and functions is critical for reducing PEDV-induced mortality rates (which can be as high as 100%) in neonatal pigs.

Although strengthening of the intestinal epithelial tight junctions and regulation of intestinal epithelium regeneration have been extensively studied with the goal of combating enteric pathogen infection [3,4], the roles of the extracellular matrix (ECM) secreted by enterocytes in maintaining and enhancing the intestinal mucosal barrier remain to be fully elucidated. Formed by a complex assortment of glycoproteins and proteoglycans, the ECM not only serves as a scaffold for intestinal tissues but also constitutes the host’s cellular microenvironment in which most infection-related events occur [5,6]. Additionally, the ECM serves as a reservoir for various signals that are components of immunological pathways. In fact, recent studies have shown that mucosal ECM components serve as first-line contacts or barriers, between host cells and pathogens [7]. Specifically, during pathogen infection, the ECM conveys specific signals to connective cells, inducing multiple immune protective effects, including antimicrobial and antiviral responses, pathogenic microorganism recognition, immune cell activation, and transcriptional regulation of inflammatory factors [8–10].

The ECM functions by interacting with neighboring cell surface receptors, a phenomenon that ultimately determines the complex cellular behaviors. Evidence has emerged that a series of classic membrane receptors are involved in ECM-modulated essential immune responses [11]. Among them, cluster of differentiation 44 (CD44) is a multidomain signaling protein that integrates ECM cues and regulates many biological processes, including cell adhesion, migration, and proliferation [12]. As an important transmembrane receptor, CD44 exhibits broad expression on the surfaces of hematopoietic, epithelial, endothelial, and mesenchymal cells and interacts with myriad ECM components, including hyaluronic acid (HA), collagen, and fibronectin, as well as numerous growth factors and cytokines [13]. Although there is clear evidence suggesting the importance of investigating the function of CD44 as a mucosal immune factor in the ECM, its specific role in protection against invading pathogens remains unclear.

During pathogen-induced ECM remodeling, the generation and degradation of ECM components are regulated by various factors. Following degradation by specific lyases, biologically active fragments of ECM proteins, such as growth factors and cytokines, are released into the extracellular microenvironment, thereby contributing to the activation of defense mechanisms in neighboring cells [14]. The fibrinolytic system, including plasminogen activators (PAs), plasminogen, plasmin, and plasminogen activator inhibitor-1 (PAI-1), have been shown to critically affect ECM degradation. The urokinase/tissue plasminogen activator (uPA/tPA) regulates the fibrinolytic system, converting zymogen plasminogen as plasmin, which triggers a proteolytic cascade [15,16]. However, the activation of uPA/tPA is inhibited by PAI-1, a serine protease inhibitor that directly titrates the overall pericellular proteolytic balance [17]. Moreover, a recent report identified extracellular PAI-1 as an unconventional interferon-stimulated protein that blocks the maturation of influenza A virus (IAV) [18]. However, it is unclear whether PAI-1 is also associated with infection with other viruses that require extracellular protease-driven maturation.

Here, we explored the functions and mechanisms of ECM components, including CD44 and PAI-1, as well as their downstream pathways, in PEDV infection. Our findings provided insights into the applications of the ECM in resisting intestinal pathogenic infections and improved our understanding of the mucosal immune system.

## Materials and methods

### Cells, antibodies, and reagents

Vero E6 cells were cultured in Dulbecco’s modified Eagle’s medium (DMEM) containing 10% fetal bovine serum (FBS) at 37°C with 5% CO_2_. Mouse monoclonal antibodies (mAbs) targeting PEDV nucleocapsid (N) protein were purchased from Median Diagnostics (South Korea). Additionally, antibodies against collagen alpha 1 (IV) (COL4A1; Abcepta; cat. no. AP7369), laminin beta 2 (LAMB2; Abcepta; cat. no. AP6836b), integrin alpha 6 (ITG6; Proteintech; cat. no. 27,189-1-AP), and decorin (DCN; Affinity; cat. no. DF6543) were used to detect changes in ECM composition. To quantify the expression of CD44 and PAI-1 proteins, anti-pig CD44 (cat. no. ab157107) and PAI-1 (cat. no. ab66705) mAbs were purchased from Abcam (Cambridge, UK). The nuclear factor (NF)-κB pathway was assessed using anti-pig p65 (cat. no. 6956) and anti-pig phospho-p65 (cat. no. 3033) antibodies from Cell Signaling Technology (Danvers, MA, USA). BAY 11–7082 (an NF-κB inhibitor) was purchased from Selleck Chemicals (China). Secondary antibodies used for immunofluorescence included goat anti-rabbit Alexa Fluor 594 (R37117), goat anti-rat Alexa Fluor 594 (cat. no. A48264), and goat anti-mouse Alexa Fluor 488 (cat. no. A-11029), purchased from Invitrogen (Carlsbad, CA, USA). Nuclei were stained with diamidino-2-phenylindole (DAPI) from Thermo Fisher Scientific (USA).

### PEDV infection

The PEDV strain Zhejiang08 was isolated from piglets exhibiting serious diarrhea in 2012; this strain was clustered with an emerging virulent strain [19]. Briefly, a cultured confluent monolayer of Vero E6 cell was subjected to 1-hour inoculation with PEDV at a multiplicity of infection (MOI) of 0.1 at 37°C. After washing, cells were cultured in DMEM supplemented with 2% FBS at 37°C in an atmosphere containing 5% CO_2_. When a cytopathic effect (CPE) of 80% was detected, cell mixtures were subjected to freezing and thawing to harvest the viruses. The harvested viruses were used for further propagation or stored at −80°C. The intestinal samples acquired from piglets orally infected with PEDV, which were used to validate the results from *in vitro* experiments, were preserved as described for a previous PEDV challenge experiment [20].

### RNA preparation and transcriptome sequencing

Using TRIzol reagent (Qiagen, MD, USA), total RNA was harvested from infected and uninfected Vero cells, and cDNA was synthesized using an HiScript II 1st Strand cDNA Synthesis Kit (Vazyme, China), in accordance with the manufacturer’s instructions. A Nano 6000 Assay Kit and NanoPhotometer spectrophotometer (IMPLEN) with the Bioanalyzer 2100 system (Agilent Technologies) were used to assess the purity and integrity of RNA.

RNA samples (3 μg) were used to generate a sequencing library using a NEBNext Ultra RNA Library Prep Kit (Illumina, NEB, USA), according to the manufacturer’s protocols, and index codes were used to attribute sequences to the appropriate samples. The library preparations were then subjected to sequencing using an Illumina Hiseq 2000 system, and 100-basepair paired-end reads were obtained. The reference genome and gene model annotation were obtained from ftp://ftp.ensembl.org/pub/. An index pertaining to the reference genome was constructed using Bowtie v2.0.6, and clean paired-end reads were aligned to the reference genome using TopHat v2.0.9. HTSeq v0.6.1 was used to determine the RPKM of respective genes based on gene lengths and read counts, with mapping to the appropriate genes. Differential expression of genes was evaluated for two scenarios/cohorts (with two biological replicates per scenario) using DESeq R package (1.10.1). Data were deposited in the Sequence Read Archive, under the accession number PRJNA679356.

### Single nucleotide polymorphism (SNP) and alternative splicing analysis

Using Asprofile v1.0 software, alternative splicing (AS) events were classified into 12 basic types. The number of AS events in respective samples was estimated separately. SNP analysis was performed using Picard-tools v1.96 and Samtools v0.1.18 for sorting, identifying copied reads, and reordering bam alignment results. GATK2 was used for SNP calling.

### Functional analysis of differentially expressed genes (DEGs) and analysis of protein-proteininteractions (PPIs)

DEGs were assessed by Gene Ontology (GO) enrichment analysis. GO terms with corrected *P* values < 0.05 were considered significantly enriched in DEGs. For analysis of the gene pathway network, we used KOBAS to test the enrichment of DEGs in Kyoto Encyclopedia of Genes and Genomes (KEGG) pathways. PPI networks among the DEGs were analyzed using the STRING database (http://string-db.org/), which included direct and indirect associations of proteins. Cytoscape was used to build the DEG network.

### Plasmid construction and lentivirus packaging

The coding sequences of *CD44* (XM_008002067.1) and serpin family E member 1 (*SERPINE1*, encoding PAI-1; XM_008018478.1) were amplified and inserted into the EcoRI/BamHI site of the pLVX-DsRed-Monomer-N1 vector.

For *CD44* and *SERPINE1* knockdown, the shRNA sequences were designed by BLOCK-It RNAi Designer (http://rnaidesigner.lifetechnologies.com/rnaiexpress/insert.do; Life Technologies; Table S1) and inserted into pLVX-shRNA1 vector (TaKaRa, China).

Lentiviral production was carried out using the Lenti-X HTX Packaging Mix (plasmids pLP1, pLP2, and VSV-G) and X-tremeGENE HP DNA Transfection Reagent (Roche, Germany), in line with the lentivirus packaging protocol. After collection, the lentivirus (MOI = 2) was added into Vero E6 cells with polybrene (8 µg/mL) for 8 h. At 48 h post-infection, puromycin was used for positive selection (8 µg/ml, Sigma-Aldrich), this experiment continued for 2 weeks to generate stable cell lines. Cell lines expressing the CD44, PAI-1, and control (scrambled) shRNA vectors were designated shRNA-CD44, shRNA-PAI-1, and shRNA-control, respectively.

### Reverse transcription quantitative polymerase chain reaction (RT-qPCR)

Cellular RNA was extracted using RNAiso Plus (TaKaRa) and was reverse transcribed using a HiScript III 1st Strand cDNA Synthesis Kit (Vazyme). Real-time PCR was performed using a TB Green PCR kit (TaKaRa). All primers used for RT-qPCR are presented in Table S2. The expression of cellular glyceraldehyde 3-phosphate dehydrogenase (*GAPDH*) was quantified as the internal control.

### Western blotting

Samples were lysed using RIPA lysis buffer, and protein concentrations were determined using BCA assay. Proteins were separated by sodium dodecyl sulfate polyacrylamide gel electrophoresis on 12% gels and transferred onto polyvinylidene difluoride membranes (Millipore, USA). After blocking with 5% skim milk, the membranes were probed with specific primary antibodies. GAPDH was used as a loading control. Protein bands were detected, and the relative protein levels were quantified using ImageJ (National Institutes of Health, Bethesda, MD, USA).

### Immunofluorescence assay (IFA)

Vero E6 cells were seeded on coverslips placed in 24-well plates and then infected with PEDV (MOI = 0.1) for 24 h. After fixation with 4% paraformaldehyde, permeabilization with 0.1% Triton X-100, and blocking with 0.1% bovine serum albumin, the cells were probed with anti-PEDV N (nucleocapsid), anti-CD44, anti-PAI-1, anti-p65, and anti-phospho-p65 antibodies. Subsequently, the cells were incubated with the corresponding secondary antibodies, and nuclei were stained with DAPI. Finally, cells were visualized using a Zeiss LSM710 confocal microscope and analyzed with ZEN 2012 software.

### Isolation and culture of porcine intestinal epithelial cells

Porcine intestinal epithelial cells (IECs) were isolated from the jejunum as previously described [21,22]. Briefly, thoroughly flush the jejunum with ice-cold sterile saline to remove its lumen contents and cut longitudinally. Rinse the lumen 3 times with Dulbecco’s Phosphate Buffered Saline (DPBS) containing 20 mM HEPES (pH 7.4) and 5 mM EDTA (disodium). The tissues were cut into small pieces (approximately 2–3 mm3) and transferred to a 50 ml tube containing 40 ml DPBS containing 20 mM HEPES (pH 7.4) and 5 mM EDTA (disodium), and rinsed three times. Then, the tissue pieces were incubated with DPBS containing 20 mM HEPES (pH 7.4) and 5 mM EDTA (disodium) at 37°C in a shaking water bath (70 shakes/min) for 30 minutes. After incubation, the tissue pieces were gently tapped and the cell suspension was filtered through a metal mesh with a pore size of 200 μm and centrifuged at 300 g at 4°C for 5 min, then washed three times with DPBS containing 20 mM HEPES (pH 7.4) without EDTA, and resuspended. The viability of the prepared enterocytes was assessed by trypan blue exclusion and the resulting cells were used for the subsequent studies.

### Plaque assay

Confluent monolayers of Vero E6 cells in 6-well tissue culture plates were infected with PEDV suspensions (500 μL of serial 10-fold dilutions) at 37°C in an atmosphere containing 5% CO_2_. After incubation for 1 h at 37°C, cells were overlaid with 0.8% low-melting-point agarose in DMEM containing 2% FBS and incubated at 37°C for 3 days. The plaques were the visualized using Crystal Violet staining.

### Statistical analysis

Statistical analyses were performed using SPSS 15.0 (SPSS Inc., Chicago, IL, USA). Data were expressed as means ± standard deviations (SDs) and RT-qPCR and western blotting data were analyzed using analysis of variance or *t*-tests. For all analyses, results with *p* values < 0.05 were considered significant. All results were obtained from triplicate experiments, unless otherwise noted.

## Results

### Characterization of DEGs following PEDV infection

To monitor the kinetics of PEDV propagation in Vero E6 cells, the CPE and viral protein were determined at different times. Although minimal CPE was visible 24 h post-infection (hpi), evident CPE was observed at 36 hpi, and nearly complete cell death was detected at 48 hpi (Figure S1a). Additionally, high levels of viral protein expression (Figure S1b) and major accumulation of intracellular virions (Figure S1c) were observed at 24 hpi. The kinetics of PEDV propagation in Vero cells was assessed by detecting viral titers and RNA in the indicated time (Figure S1d and S1e). Growth-kinetic studies showed that the virus replicated rapidly and efficiently in Vero E6 cells with an almost logarithmic growth form from 12 h to 24 h, reaching a maximum titer of 10^6^ PFU/ml by 48 hpi.

To ensure a high proportion of infected cells while avoiding excessive CPE, mock- or PEDV-infected cells were collected 24 hpi for RNA-seq. This analysis identified 14,715 and 13,544 genes in the control and PEDV-infected groups having an RPKM greater than 1, respectively, and Venn diagram analysis (Figure S2a) identified 1793 genes expressed only in the control group and 622 genes expressed exclusively in the PEDV-infected group. The reduced gene expression in the PEDV group indicated that the virus may influence functional gene expression in the infected Vero cells. To further investigate the differential expression patterns in Vero cells, genes with a |log_2_ (fold change)| greater than 1 and *p*_.adj_ less than 0.001 were considered significant DEGs. Automatic offset correction was used to reduce manual error. Ultimately, 5647 DEGs were identified and their expression was quantified in PEDV-infected cells. Among these genes, 2898 genes exhibited significant upregulation, whereas 2739 genes exhibited marked downregulation during PEDV infection (Figure S1b and Data S1).

SNPs and insertion/deletion (InDel) events from the PEDV-infected and control samples were also characterized. Among the identified SNP transversions, A/G, G/A, C/T, and T/C transitions exhibited the highest abundances, whereas A/T and T/A transitions were the least abundant (Figure S2c). Furthermore, putative SNPs and InDel events decreased after PEDV infection. Among the 12 basic types of AS events, the transcriptional start site, transcription termination site, and skipped exons were the primary events observed following PEDV infection (Figure S2d). The expression levels of six DEGs determined by RNA-seq were verified by RT-qPCR. The result showed that the expression levels of these six DEGs were consistent with those determined by RNA-seq (Figure S2e).

### PEDV infection disrupted ECM expression

**Figure 1.**
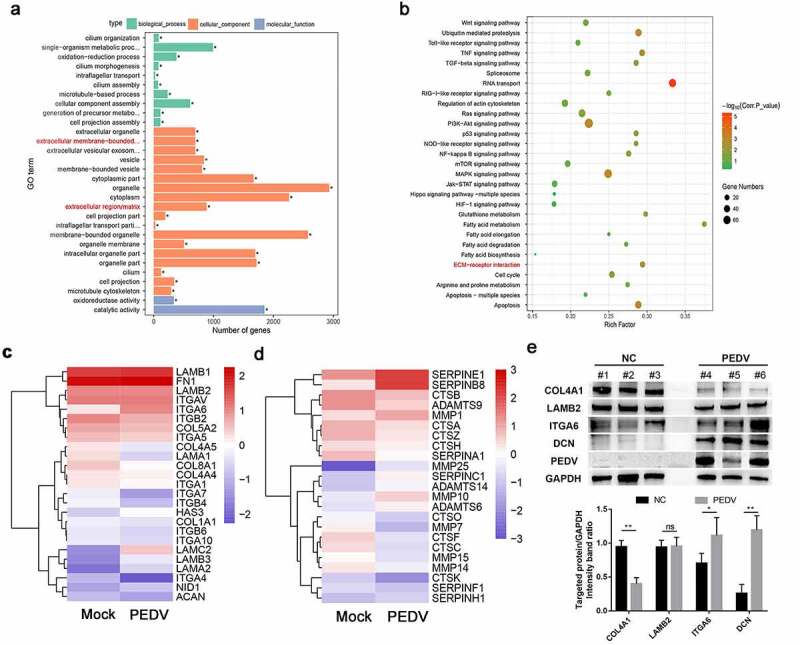
Enrichment analysis and expression profiles of differentially expressed genes (DEGs). (a) Functional map for downregulated DEGs enriched for GO terms. (b) Statistical analysis of the top 30 enriched KEGG pathways during PEDV infection. The x-axis indicates the factor of enrichment, whereas the y-axis indicates the KEGG channel. The dot color indicates the *q* value. The dot size corresponds to the number of DEGs mapped to the reference category. (c and d) Hierarchical clustering of extracellular matrix-constituent (c) and -regulated (d) DEGs. Rows indicate hierarchical clustering in accordance with average linkage based on Pearson correlation coefficients with the measured distance. Expression was visualized using a gradient color scheme; the scale from the minimal to maximal abundance fell in the range of −3.0 to 3.0. Blue represents low expression, whereas red represents high expression. (e) The total protein collected from the small intestine of PEDV-infected piglets (48 h post-infection) was prepared, and the expression levels of ECM-associated proteins (i.e., COL4A1, LAMB2, ITG6, and DCN) and PEDV N protein were determined by western blotting. The data shown are means ± SDs from triplicate experiments. *P < 0.05, **P < 0.01

To identify the roles of the obtained DEGs and annotate the genes to their biological, cellular, and molecular functions, we performed GO analysis using GOseq R package. Most of the downregulated DEGs were classified into the cellular components category, in which ECM and ECM-bound organelle categories were highly enriched (Figure 1(a)). Furthermore, DEGs were investigated to using KEGG analysis to identify biological pathways enriched during infection. In addition to canonical signaling pathways (e.g., RNA transport, mitogen-activated protein kinase, tumor necrosis factor, and NF-κB, all of which are associated with PEDV infection), the ECM-receptor interaction pathway was also identified through KEGG enrichment analysis (Figure 1(b)). Heat map analysis identified DEGs involved in ECM construction and modulation. Compared with the mock group, the PEDV-infected group exhibited significant up- or downregulation of many ECM components (e.g., *LAMA, LAMB, ITGB, ITGA, COL1A, HSA3*, and *COL8A1*) and ECM regulating molecules (e.g., *SERPINE, MMP25, MMP7, CTSC*, and *CTSB*; Figure 1(c,d)). Furthermore, significant changes in the expression of genes encoding ECM component proteins, including *COL4A1, DCN*, and *ITGA6*, were also identified in the small intestine of PEDV-infected piglets (Figure 1(e)).

### Correlational analysis between ECM-related genes and PEDV infection

**Figure 2.**
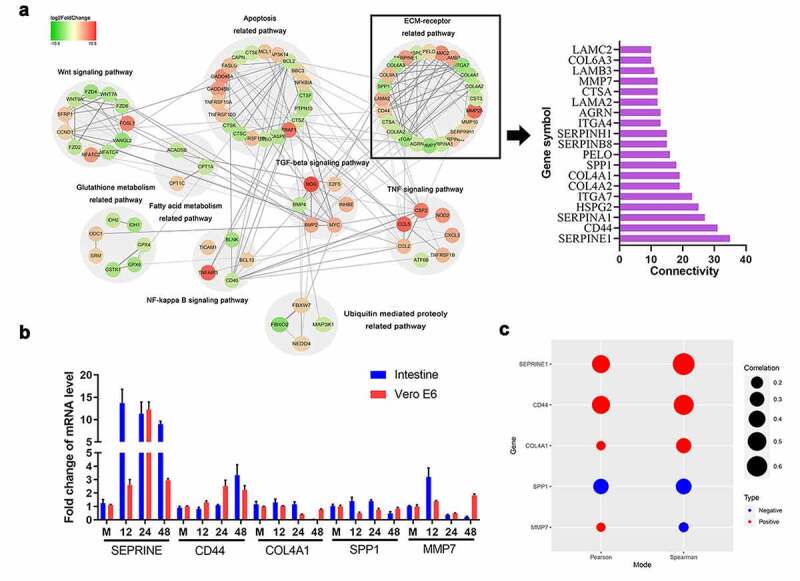
Protein-protein interaction (PPI) network analysis and evaluation of the relationships between the DEGs and ECM receptor signaling. (a) A PPI network of DEGs was constructed using STRING and visualized using Cytoscape. The hub genes in the bar chart were identified based on the connectivity degree within the PPI. DEGs are represented as round nodes. Green and red nodes indicate downregulated and upregulated DEGs, respectively. Proteins with mutual associations show edge-based connections, and combined interaction scores are indicated by the edge thickness. (b) The mRNA levels of hub genes in Vero E6 cells and intestinal tissues infected with PEDV were quantified using RT-qPCR. (c) Correlation analysis between the expression of hub genes in Vero E6 cells and intestinal tissues infected with PEDV. The color of the dots indicates positive or negative correlations, and the dot size indicates the correlation coefficient (r); Pearson and Spearman correlation analyses were employed for determining the r value. Data are presented as means ± SDs of three independent experiments. *P < 0.05, **P < 0.01

Based on the KEGG results, 85 DEGs (from the top 1000 up- or downregulated genes) from nine activated signaling pathways were selected for the construction of a PPI network. The Cystoscape software was visualized the protein interaction networks of the DEG products, while the connective degree of the DEGs was calculated by plugin CytoHubba (Figure 2(a)). The hub DEGs (connective degree > 15) involved in the ECM receptor-related pathway were presented as a histogram. Based on the functional annotations, we further detected the dynamic expression of five highly connected DEGs (i.e., *SERPINE, CD44, COL4A1, SPP1*, and *MMP7*) in Vero E6 cells and intestinal tissues from PEDV-infected piglets (Figure 2(b)). Pearson and Spearman correlation analysis further identified *SERPINE1* and *CD44* as being significantly correlated with PEDV replication, indicating that these genes may influence ECM construction and signal transduction during PEDV infection (Figure 2(c)).

### PEDV altered CD44 and PAI-1 protein expression

**Figure 3.**
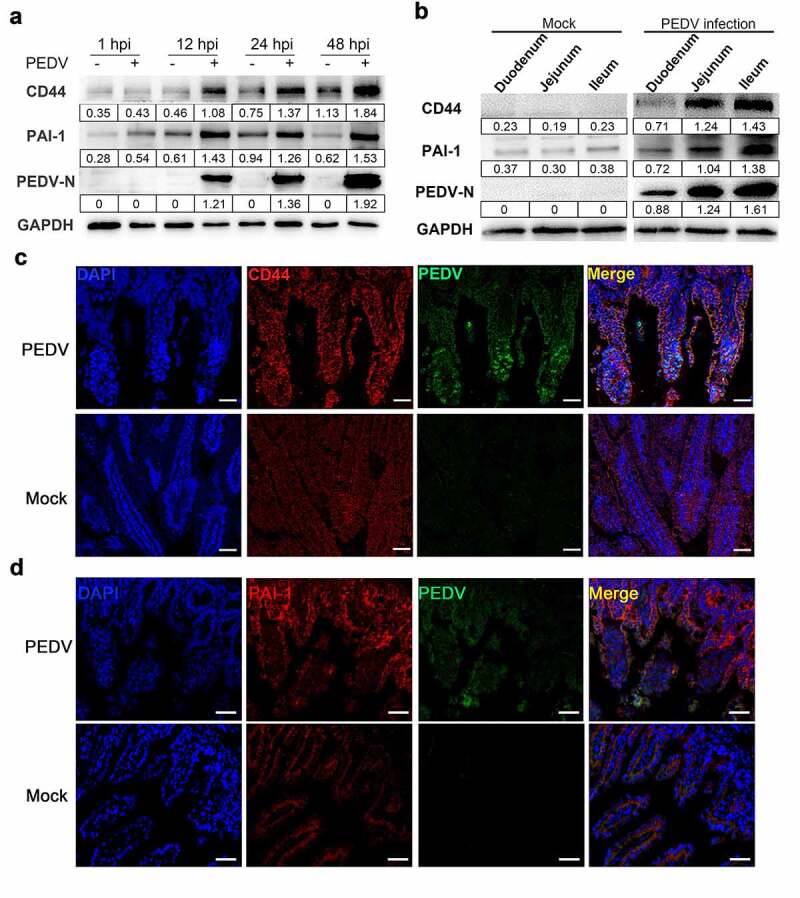
Infection with PEDV enhanced CD44 and PAI-1 protein levels. Vero E6 cells were mock-infected or PEDV-infected (MOI = 0.1) at the time indicated (1–48 h), then cellular total proteins were harvested. (a) CD44 and PAI-1 protein levels in Vero E6 cells were determined by western blotting. GAPDH was taken as the internal reference; the ratio of each protein gray value to that of GAPDH was regarded as the relative expression of protein. (b) Total protein was isolated from the intestinal tissues of PEDV-infected piglets (48 hpi), and CD44, PAI-1, and PEDV protein levels in the duodenum, jejunum, and ileum were detected by western blotting. (c and d) Staining for PAI-1 (d), CD44 (c), and PEDV antigen (N protein) based on immunofluorescence in the jejunum of PEDV-infected piglets. Red, CD44 or PAI-1; green, PEDV; blue, DAPI. Scale bars, 25 μm. The data shown represent means ± SDs from triplicate experiments. *P < 0.05, **P < 0.01

To investigate the expression of CD44 and PAI-1 (encoded by SERPINE1) during PEDV infection, we extracted total protein from mock-infected or PEDV-infected Vero E6 cells (MOI = 0.1) at the indicated times (1–48 h). Compare to mock-infected group, the quantity of intracellular CD44 and PAI-1 protein showed an increasing trend after viral infection. At 12 or 24 h after infection, the abundances of CD44 and PAI-1 were nearly 2-fold higher than those of the controls (Figure 3(a)). To validate in porcine cells, primary porcine intestinal epithelial cells (IECs) were isolated from one-week-old piglets. After PEDV inoculation, the protein and mRNA levels of CD44 and PAI-1 in IECs were up-regulated (Figure S3), which exhibited similar expression change as that in Vero E6 cells. Analysis of the expression of CD44 and PAI-1 in response to PEDV infection in piglets revealed that these molecules were highly upregulated in different segments of the small intestine in PEDV-infected piglets (compared with that in mock-infected controls); this upregulation correlated with viral loading (Figure 3(b)). Moreover, IFA revealed high abundances of CD44 and PAI-1 in the jejunum of PEDV-infected piglets and showed that these proteins mainly accumulated in intestinal epithelial cells (Figure 3(c,d)).

### CD44 hampered PEDV infection of host cells

**Figure 4.**
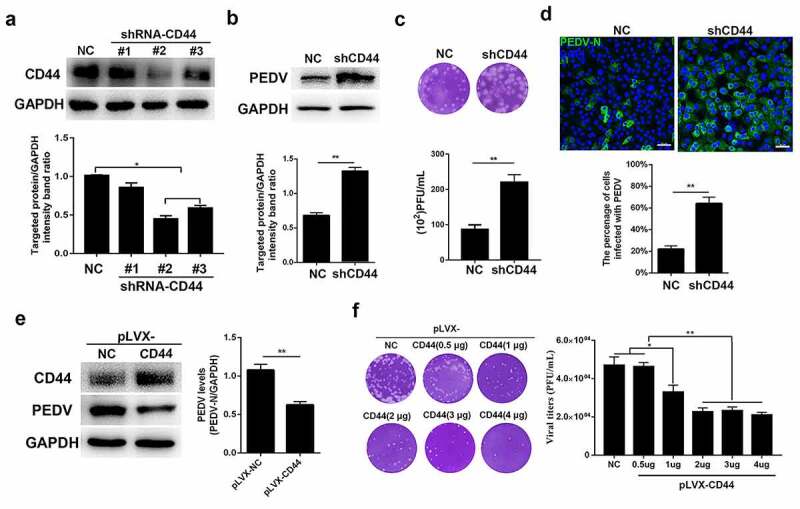
CD44 impeded PEDV infection. (a) CD44 knockdown verification. Cells were transfected with three shRNAs targeting CD44, and the silencing efficiency was verified by western blotting. Subsequently, Vero E6 cells stably transfected with shRNA-CD44#2 were established and inoculated with PEDV for 24 h. (b) PEDV protein levels in CD44-silenced cells were detected by western blotting. (c) Virus titers in the supernatants of cultures of CD44-silenced cells were detected by plaque assays. (d) Staining for PEDV antigen (N protein) in mock-transfected cells and CD44-silenced cells was evaluated by immunofluorescence. Green, PEDV; blue, DAPI. Bars, 50 μm. (e) Increased CD44 expression inhibited PEDV infection. Vero E6 cells were transfected with lentiviral particles to generate CD44-overexpressing cells and then infected with PEDV for 24 h. Western blotting was performed to determine the expression levels of CD44 and PEDV N proteins. (f) Vero E6 cells were transfected with a CD44 expression vector (pLVX-CD44) and infected with PEDV for 24 h. The extracellular virus titers were measured using plaque assays. The data represent means ± SDs of triplicate assays. * *P* < 0.05; ** *P* < 0.01

As a transmembrane receptor protein, CD44 forms a connection between the ECM and neighboring cells. To assess whether CD44 influenced PEDV infection, Vero E6 cells were used to generate stable knockdown of CD44 expression by transfection with shRNA vectors. Compared with cells transfected with shRNA-NC, the expression of CD44 was decreased significantly in cells transfected with shRNA-CD44#2 and shRNA-CD44#3 (Figure 4(a)). Subsequently, the replication characteristics of PEDV (MOI = 0.1) were detected in shRNA-CD44#2-transfected Vero cells. As shown in Figure 4(b,c), knockdown of CD44 expression strongly promoted viral protein expression and progeny yield in cells following PEDV infection, as compared with that in shRNA-NC-transfected cells. Furthermore, the effects of CD44 on PEDV infection were visualized by IFA. In comparison with mock-transfected cells, shRNA-CD44#2-transfected cells showed greater PEDV positivity (Figure 4(d)).

For additional verification of the effects of CD44 on PEDV infection, Vero E6 cells were transfected with the recombinant plasmid pLVX-DsRed harboring the green monkey *CD44* gene. At 72 h after transfection, cells were infected with PEDV (MOI = 0.1) for 24 h. Successful overexpression of CD44 was confirmed by western blotting, resulting in a nearly 50% reduction in PEDV protein expression (Figure 4(e)). Additionally, plaque formation revealed that CD44 significantly decreased PEDV titers in a dose-dependent manner (Figure 4(f)). Nevertheless, CD44 knockdown and overexpression did not impact cell viability, as demonstrated using Cell Counting Kit 8 proliferation assays (Figure S4).

### CD44 blocked PEDV infection by activating the inflammatory pathway

**Figure 5.**
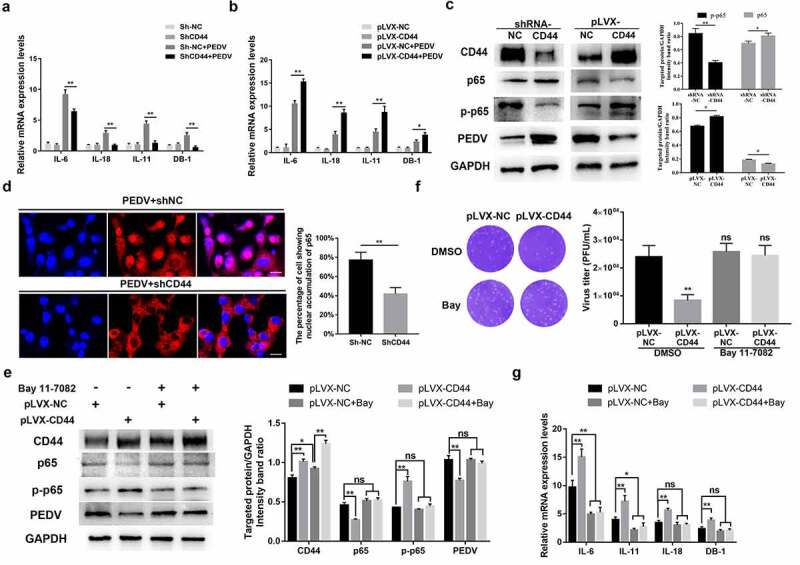
CD44 upregulated antiviral cytokines by activating NF-κB signaling. (a) CD44 knockdown blocked the secretion of antiviral cytokines following PEDV infection. CD44-knockdown and mock-transfected Vero E6 cells were infected with PEDV (MOI = 0.1) for 24 h, and *IL-18, IL-11*, and *BD-1* mRNA levels were quantified by RT-qPCR. (b) CD44 overexpression enhanced antiviral cytokine secretion. *IL-18, IL-11*, and *BD-1* mRNA levels were detected in CD44-overexpressing Vero E6 cells. (c) CD44 induced the phosphorylation of NF-κB in Vero E6 cells. Cells were infected with PEDV (MOI = 0.1) for 24 h, and CD44, p65, phospho-p65, PEDV (N protein), and GAPDH (loading control) protein levels in CD44-knockdown and -overexpressing cells were detected by western blotting. Statistical results for p65 and phospho-p65 are shown in bar charts. (d) CD44-knockdown cells were infected with PEDV, and the nuclear translocation of p65 was verified by IFA. Colors correspond to nuclei (blue) and p65 (red). Scale bars, 25 μm. Nuclear accumulation of p65 was evaluated in 200 cells in three independent experiments; the data are shown in bar charts. (e–g) To inhibit NF-κB activation, CD44-overexpressing Vero E6 cells were treated with BAY 11–7082 (10 μM) or DMSO for 2 h at 37°C. After washing, cells were infected with PEDV (MOI = 0.1) for 24 h and harvested for subsequent analyses. (e) The protein levels of CD44, p65, phospho-p65, PEDV (N protein), and GAPDH were detected by western blotting. Statistical results for CD44, p65, phospho-p65, and PEDV are presented in bar charts. (f) virus titers were detected by plaque assays. (g) *IL-18, IL-11*, and *DB-1* mRNA levels were further determined. The data indicate means ± SDs of triplicate assays. * *P* < 0.05; ** *P* < 0.01

Inflammatory cytokines elicit widespread innate immune responses and enhance the host’s defense against pathogen infections [23,24]. Previous studies have demonstrated that CD44 is involved in cytokine regulation. To detect whether the antiviral effects induced by CD44 were associated with inflammatory cytokine expression, we assessed the transcript-levels of cytokines in stable shRNA-CD44- and Plvx-CD44-transfected Vero E6 cells. Overexpression of *CD44* resulted in enhanced expression of *IL-6, IL-18, IL-11*, and defensin beta I (*DB-1*) following PEDV infection, whereas in CD44-knockdown cells, the transcript-level of these cytokines was significantly decreased (Figure 5(a,b)).

NF-κB signaling is a central coordinating signal for the expression of many inflammatory cytokines and host innate immune responses [25]. Moreover, phosphorylation and nuclear translocation of p65 are critical indicators of NF-κB signal activation [26]. Hence, following PEDV infection (MOI = 0.1), the levels of p65 and phospho-p65 were also detected in CD44-overexpressing and knockdown cell lines (Figure 5(c)). Compared with mock-transfected Vero E6 cells, overexpression of CD44 increased the phosphorylation of p65. However, the activation of p65 was significantly inhibited in CD44-knockdown Vero cells during PEDV infection. Additionally, the nuclear translocation of p65 was verified by indirect IFA (Figure 5(d)), further verifying that the nuclear translocation of p65 induced in response to PEDV infection was partial abrogated (50%) in CD44-knockdown Vero E6 cells. Moreover, pretreatment of CD44-overexpressed cells with an inhibitor of NF-κB (BAY 11–7082) significantly counteracted the inhibitory effects of CD44 on PEDV infection (Figure 5(e,f)), as well as the secretion of antiviral cytokines induced by CD44 (Figure 5(g)). In contrast, the cell viability of each group was not influenced by inhibitor treatment (Figure S6).

### Role of PAI-1 protein in PEDV infection

**Figure 6.**
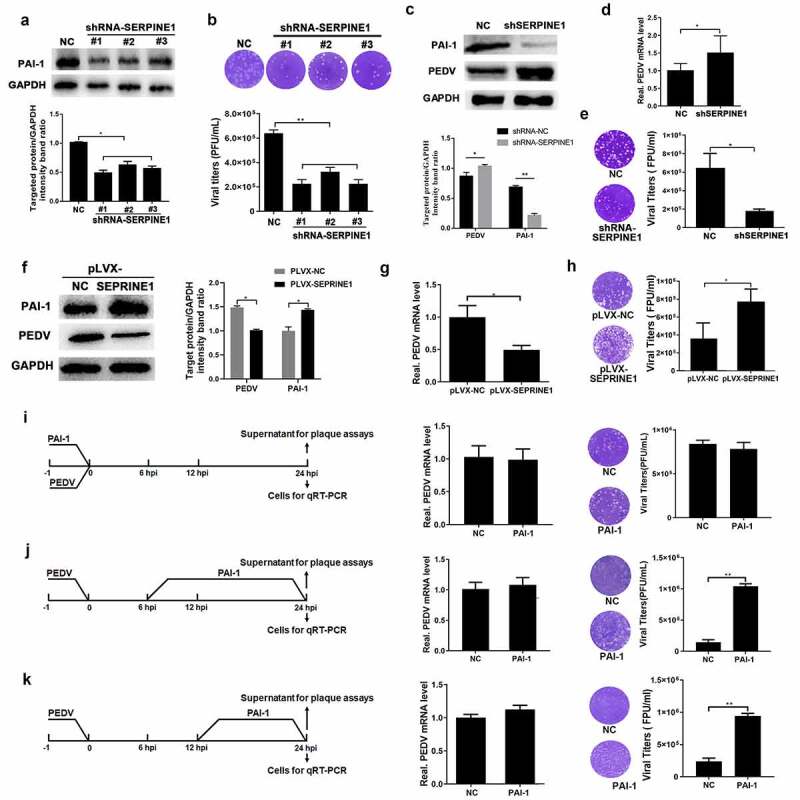
PAI-1 promoted PEDV release. (a) PAI-1 knockdown verification. Cells were infected with lentivirus particles expressing shRNAs targeting *SERPINE1*. After 48 h, western blotting was performed to investigate *SERPINE1* knockdown efficiency. (b) Following PEDV infection (MOI = 0.1), extracellular virus titers were evaluated using plaque tests. (c–e) *SERPINE1*-knockdown cell lines were established by puromycin screening, and greater than 80% interference efficiency was achieved. (c) *SERPINE1*-knockdown cell lines were infected with PEDV (MOI = 0.1) for 24 h, and PEDV N expression was evaluated by western blotting. (d) Viral RNA expression was evaluated by RT-qPCR. (e) Extracellular virus titers were measured using plaque assays. (f) PAI-1-overexpressing cell lines were infected with PEDV (MOI = 0.1) for 24 h, and western blotting was performed to investigate PAI-1 and PEDV N expression. (g) Viral RNA expression was quantified by RT-qPCR. (h) The yield of the progeny virus was measured using plaque assays. Extracellular PAI-1 affected the replication stage of PEDV infection. PAI-1 (160 ng/mL) was added to the medium at the entry (−1 h to 0 h of inoculation) (i), prebudding (6–24 hpi) (j), or release (12–24 hpi) (k) stages of PEDV infection (MOI = 0.1). Intracellular viral RNA and extracellular virus titers were detected at 24 h after PEDV inoculation. The data represent means ± SDs of triplicate assays. * *P* < 0.05; ** *P* < 0.01

Vero E6 cells were transiently transfected with shRNAs targeting *SERPINE1*, and the interference effect of the shRNA was confirmed by investigating PAI-1 expression (Figure 6(a)). Compared with control shRNA (scrambled) transfection, suppression of *SERPINE1* expression significantly inhibited the viral progeny yield in PEDV-infected Vero cells (Figure 6(b)). Additionally, we generated Vero E6 cells exhibiting stable downregulation of *SERPINE1* (> 70% inhibition efficiency), and the cells were subsequently infected with PEDV (MOI = 0.1). Although the release of the viral progeny was inhibited in *SERPINE1*-knockdown cells (Figure 6(e)), the expression of viral protein (Figure 6(c)) and mRNA was unexpectedly increased (Figure 6(d)).

For further investigation of the effects of PAI-1 in PEDV infection, we transfected Vero E6 cells with pLVX- SERPINE1 and confirmed the overexpression efficiency of PAI-1 (Figure 6(f)). After PEDV infection (MOI = 0.1), the protein and mRNA levels of PEDV genes in PAI-1-overexpressing cells were significantly decreased compared with those in mock-transfected cells (Figure 6(f,g)). However, the number of infectious viral virions released from cells increased by 50% (Figure 6(h)). Cell viability assays showed that recombinant PAI-1 did not elicit any significant cytotoxic effects at concentrations of 5–200 ng/mL (Figure S5). According to the recommended working concentration in manual, and the growth-kinetics for PEDV Zhejiang08, recombinant PAI-1 at a concentration of 160 ng/mL was added to the medium of Vero E6 cells at the stage of PEDV entry (−1 hpi-0 hpi), prebudding (6 hpi-24 hpi), release (12–24 hpi). Subsequently, the culture supernatant and cellular samples were harvested at 24 hpi, and analyzed to quantify the infectious particle titers, as well as the levels of viral RNA. Notably, treatment with PAI-1 during the viral entry stage did not affect the quantity of intracellular viruses or infectious virion release (Figure 6(i)). However, treatment of cells with PAI-1 during the viral prebudding or release stage resulted in effects, significantly promoting the release of infectious viral progeny, although no effects on intracellular viral levels were detected (Figure 6(j,k)).

## Discussion

Because the primary pathological characteristic of PEDV infection is severe intestinal mucosal injury, it is critical to maintain and enhance the integrity of the intestinal mucosal barrier to effectively reduce the risk of mortality associated with PEDV [27]. As the primary component of the microenvironment in the intestinal mucosa, the ECM serves as a physical support for tissue integrity and elasticity [28]. Indeed, during infection, the various and specific effects of the ECM alter the invasion and spread of microbial pathogens in host tissues and significantly affect the overall immune response to infection [29,30].

During pathogenic infection, host cells are constantly rebuilding and remodeling the ECM to limit pathogen infection and restrict disease progression [14]. This phenomenon is observed in heparin-binding peptides derived from laminin, vitronectin and fibronectin, which exert a direct inhibitory effect on bacterial infection [31]. Moreover, the extracellular protein Tenascin-C has been reported to reduce human immunodeficiency virus (HIV) infection by its neutralizing activity [32]. Notably, the ECM and innate immune response are inextricably linked. During pathogenic infection, the ECM transmits specific signals that feed into immunological and inflammatory pathways, thereby modulating essential immune functions within infected tissues [7,33]. Such as mindin (a member of secreted ECM proteins) which induces the synthesis of a series of pro-inflammatory cytokines (e.g. TNF-α and IL-6), and the hyaluronan fragments which stimulates both inflammatory cytokine (e.g., MIP-1α, CCL-2, IL-12, and TNF-α) and IFNβ secretion via TLR-2 or TLR-4-dependent pathway [8,34,35]. Additionally, ECM cleavage enzymes known as matrix metalloproteases (MMPs) regulate innate immunity by promoting the release of pro-inflammatory cytokines, chemokines, and other immune-related proteins. For example, MMP-12 and MMP-25 mediate *IκBα* transcription in NF-κB signaling, leading to the protection of the host against coxsackievirus type B3 infection [36,37].

In the current study, we found that the expression of ECM components in the intestine was affected by PEDV infection, along with activation of ECM receptor-related pathways. To explore the potential functions of the ECM and associated downstream pathways in PEDV infection, we screened for highly connected DEGs in the ECM receptor-related pathways by PPI analysis. Our results showed that *CD44* and *SERPINE1* expression levels were positively correlated with PEDV mRNA levels. Hence, the biological functions of PEDV-induced *CD44* and *SERPINE1* expression were further investigated.

As a transmembrane glycoprotein, CD44 is a multidomain signal integration protein for several growths factor-induced and immune-regulated signaling pathways [38]. The extracellular domain of CD44 mediates interactions with several ECM molecules, including hyaluronan, collagen, fibronectin, and laminin. Recently, the association between CD44 and viral infection has attracted much attention [39]. For example, incorporation of CD44 into virions is required for the efficient dissemination of HIV-1 into secondary lymphoid organs [40]. Moreover, hepatitis C virus (HCV)-induced osteopontin promotes HCV replication and assembly by binding to αVβ3 and CD44 [41]. However, the role of CD44 in PEDV infection remains unknown. In this study, we observed significantly increased PEDV replication in *CD44*-knockdown Vero E6 cells, and infection of *CD44-*overexpressing Vero E6 cells with PEDV was significantly inhibited. Hence, our study is the first to report the role of CD44 in protecting the intestine against viral infection, suggesting that CD44 could be a potential antiviral protein for novel interventions in intestinal coronavirus infection.

The inflammatory response is the primary host defense against pathogenic infection. CD44 could act as a docking site for mediating the inflammatory response, thereby contributing to its antiviral functions [42]; however, this potential mechanism underlying CD44 antiviral activity requires further investigation. Our results showed that CD44 inhibited PEDV replication while activating the NF-κB signaling pathway. Additionally, NF-κB inhibitors reversed the inhibitory effects of CD44 on PEDV replication. Furthermore, the activation of CD44/NF-κB signaling promoted the expression of protective genes, including *IL-11, BD-1, IL-6*, and *IL-18*, in Vero E6 cells. These genes primarily act on the mucosal epithelium and exhibit robust antiviral activity within the mucosal surface [20,43–46]. Activation of NF-κB signaling is a hallmark of the cellular response to various pathogens. As a transcription factor that mediates the expression of antiviral cytokines, NF-κB is a central orchestrator of the response to viral infection [26]. Notably, NF-κB has recently been shown to support the replication of several viruses, including influenza virus [47], murine herpesvirus [48], and transmissible gastroenteritis virus (TGEV) [49]. Importantly, these viruses inhibit NF-κB activation in the early stage of infection. For example, TGEV and PEDV exhibit rapid replication with almost logarithmic growth from 6 hpi, whereas NF-κB is not activated until 24 hpi, by which time most of the host cells have already been infected [50,51]. Under such circumstances, the cytokines induced by NF-κB activation not only are unable to induce the antiviral response but also aggravate tissue damage, thereby facilitating viral spread at the site of infection.

In this study, we also explored the functions of PAI-1 in PEDV infection. As the main tissue plasminogen activator and urokinase in the ECM, PAI-1 is secreted by epithelial, endothelial, and immune cells in various tissues, including the intestines. Our findings suggested that PAI-1 promoted the generation of infectious virions at the stage of PEDV replication, which was contradictory to the current knowledge on the antiviral activity of PAI-1. Indeed, PAI-1 was recently shown to inhibit IAV maturation by targeting host proteases required for viral glycoprotein cleavage, thereby reducing the extracellular maturation of IAV particles [18]. Additionally, independent of its roles as an extracellular protease inhibitor, intracellular PAI-1 inhibits HCV replication by stimulating innate host cell defenses [52]. In our experiments, both intracellular and secreted PAI-1 increased the yield of mature PEDV virions. Therefore, as a secretory protease inhibitor, we postulate that PAI-1 may influence the activities of proteases required for viral protein translocation, assembly, and release; however, the specific mechanisms involved in this process remain unclear. Because enhancing viral release is an unexpected function of the PAI-1 protein, this protein may be a potential target for the development of novel therapies against PEDV infection.

In conclusion, we found that the ECM components of Vero E6 cells were markedly altered during PEDV infection; the ECM-regulated receptor molecules *CD44* and *SERPINE1* both exhibited high correlations with viral replication. Moreover, CD44 was identified as a host factor that inhibited PEDV infection by activating NF-κB nuclear translocation and enhancing the release of protective cytokines, thereby reducing PEDV replication in host cells. In contrast to previous studies, PAI-1, encoded by *SERPINE1*, had unexpected effects of promoting progeny PEDV virion release, suggesting a unique mechanism underlying the involvement of PAI-1 in PEDV infection. Taken together, our findings provided insights into the functions of ECM-related proteins in restricting or promoting PEDV infection, highlighting the important roles of the ECM and its downstream pathways in intestinal pathogen infection.

## Supplementary Material

Supplemental Material

## Data Availability

The authors declare that all data supporting the findings of this study are available in the manuscript and its Supplementary Information files or are available from the corresponding author upon request.

## Abbreviations

PEDV: porcine epidemic diarrhea virus; ECM: extracellular matrix; CD44: cluster of differentiation 44; SERPINE1: serpin family E member 1; DEGs: differently expressed genes; BD-1: beta-defensin 1; uPA/tPA: urokinase/tissue plasminogen activators; PAI-1: plasminogen activator inhibitor‐1; PPI: protein-protein interaction; COL4A1: collagen alpha 1 (IV); LAMB2: laminin beta 2; ITGA6: integrin alpha 6; DCN: decorin; FBS: fetal bovine serum; CPE: cytopathic effect; SNP: single nucleotide polymorphism; GO: Gene Ontology; shRNA: small hairpin RNA; hpi: hours post infection; GAPDH: glyceraldehyde 3-phosphate dehydrogenase; IFA: indirect immunofluorescence assay

Sequence information was deposited in the NCBI Sequence Read Archive (SRA) with the accession number PRJNA679356.

## References

[1] Jung K, Saif LJ, Wang Q. Porcine epidemic diarrhea virus (PEDV): an update on etiology, transmission, pathogenesis, and prevention and control. Virus Res. 2020;286:198045. 32502552 10.1016/j.virusres.2020.198045 PMC7266596

[2] Jung K, Saif LJ. Porcine epidemic diarrhea virus infection: etiology, epidemiology, pathogenesis and immunoprophylaxis. Vet J. 2015;204:134–143. 25841898 10.1016/j.tvjl.2015.02.017 PMC7110711

[3] Yu QH, Yang Q. Diversity of tight junctions (TJs) between gastrointestinal epithelial cells and their function in maintaining the mucosal barrier. Cell Biol Int. 2009;33:78–82. 18938254 10.1016/j.cellbi.2008.09.007

[4] VanDussen KL, Carulli AJ, Keeley TM, et al. Notch signaling modulates proliferation and differentiation of intestinal crypt base columnar stem cells. Development. 2012;139:488–497. 22190634 10.1242/dev.070763 PMC3252352

[5] Hynes RO. The extracellular matrix: not just pretty fibrils. Science. 2009;326:1216–1219. 19965464 10.1126/science.1176009 PMC3536535

[6] Yue B. Biology of the extracellular matrix: an overview. J Glaucoma. 2014;23:S20–3. 25275899 10.1097/IJG.0000000000000108 PMC4185430

[7] Tomlin H, Piccinini AM. A complex interplay between the extracellular matrix and the innate immune response to microbial pathogens. Immunology. 2018;155:186–201. 29908065 10.1111/imm.12972 PMC6142291

[8] He Y-W, Li H, Zhang J, et al. The extracellular matrix protein mindin is a pattern-recognition molecule for microbial pathogens. Nat Immunol. 2004;5:88–97. 14691481 10.1038/ni1021

[9] Gaudet AD, Popovich PG. Extracellular matrix regulation of inflammation in the healthy and injured spinal cord. Exp Neurol. 2014;258:24–34. 25017885 10.1016/j.expneurol.2013.11.020 PMC4099942

[10] Papageorgiou AP, Heymans S. Interactions between the extracellular matrix and inflammation during viral myocarditis. Immunobiology. 2012;217:503–510. 21907443 10.1016/j.imbio.2011.07.011

[11] Frantz C, Stewart KM, Weaver VM. The extracellular matrix at a glance. J Cell Sci. 2010;123:4195–4200. 21123617 10.1242/jcs.023820 PMC2995612

[12] Orian-Rousseau V. CD44 acts as a signaling platform controlling tumor progression and metastasis. Front Immunol. 2015;6:154. 25904917 10.3389/fimmu.2015.00154 PMC4389564

[13] Morath I, Hartmann TN, Orian-Rousseau V. CD44: more than a mere stem cell marker. Int J Biochem Biotechnol. 2016;81:166–173. 10.1016/j.biocel.2016.09.009 27640754

[14] Bonnans C, Chou J, Werb Z. Remodelling the extracellular matrix in development and disease. Nat Rev Mol Cell Biol. 2014;15:786–801. 25415508 10.1038/nrm3904 PMC4316204

[15] Collen D. The plasminogen (fibrinolytic) system. Hrombosis Haemostasis. 1999;82:259–270. 10605712

[16] Draxler DF, Medcalf RL. The fibrinolytic system-more than fibrinolysis? Transfus Med Rev. 2015;29:102–109. 25576010 10.1016/j.tmrv.2014.09.006

[17] Ghosh AK, Vaughan DE. PAI-1 in tissue fibrosis. J Cell Physiol. 2012;227:493–507. 21465481 10.1002/jcp.22783 PMC3204398

[18] Dittmann M, Hoffmann HH, Scull MA, et al. A serpin shapes the extracellular environment to prevent influenza A virus maturation. Cell. 2015;160:631–643. 25679759 10.1016/j.cell.2015.01.040 PMC4328142

[19] Li Y, Wu Q, Huang L, et al. An alternative pathway of enteric PEDV dissemination from nasal cavity to intestinal mucosa in swine. Nat Commun. 2018;9:3811. 30232333 10.1038/s41467-018-06056-w PMC6145876

[20] Li Y, Wu Q, Jin Y, et al. Antiviral activity of interleukin-11 as a response to porcine epidemic diarrhea virus infection. Vet Res. 2019;50:111. 31864417 10.1186/s13567-019-0729-9 PMC6925494

[21] Wu G, Knabe DA, Yan W, et al. Glutamine and glucose metabolism in enterocytes of the neonatal pig. Am J Physiol. 1995;268:R334–42. 7864226 10.1152/ajpregu.1995.268.2.R334

[22] Booth C, O’Shea JA. Isolation and culture of intestinal epithelial cells. Culture Epithelial Cells. 2002;2:303–335.

[23] Koyama S, Ishii KJ, Coban C, et al. Innate immune response to viral infection. Cytokine. 2008;43:336–341. 18694646 10.1016/j.cyto.2008.07.009

[24] Sartor RB, Hoentjen F. Proinflammatory cytokines and signaling pathways in intestinal innate immune cells. Mucosal Immunol. 2005;30:681–701.

[25] Oeckinghaus A, Hayden MS, Ghosh S. Crosstalk in NF-κB signaling pathways. Nat Immunol. 2011;12:695–708. 21772278 10.1038/ni.2065

[26] Deng L, Zeng Q, Wang M, et al. Suppression of NF-κB activity: a viral immune evasion mechanism. Viruses. 2018;10:409. 10.3390/v10080409 PMC6115930 30081579

[27] Zong QF, Huang YJ, Wu LS, et al. Effects of porcine epidemic diarrhea virus infection on tight junction protein gene expression and morphology of the intestinal mucosa in pigs. Pol J Vet Sci. 2019;22:345–353. 31269354 10.24425/pjvs.2019.129226

[28] Swinehart IT, Badylak SF. Extracellular matrix bioscaffolds in tissue remodeling and morphogenesis. Dev Dyn. 2016;245:351–360. 26699796 10.1002/dvdy.24379 PMC4755921

[29] Hammerschmidt S, Rohde M, Preer KT. Extracellular matrix interactions with gram-positive pathogens. Microbiol Spectr. 2019;7(2):7. 10.1128/microbiolspec.gpp3-0041-2018 PMC11590433 31004421

[30] Singh B, Fleury C, Jalalvand F, et al. Human pathogens utilize host extracellular matrix proteins laminin and collagen for adhesion and invasion of the host. FEMS Microbiol Rev. 2012;36:1122–1180. 22537156 10.1111/j.1574-6976.2012.00340.x

[31] Andersson E, Rydengård V, Sonesson A, et al. Antimicrobial activities of heparin‐binding peptides. Eur J Biochem. 2004;271:1219–1226. 15009200 10.1111/j.1432-1033.2004.04035.x

[32] Fouda GG, Jaeger FH, Amos JD, et al. Tenascin-C is an innate broad-spectrum, HIV-1–neutralizing protein in breast milk. Proc Nat Acad Sci. 2013;110:18220–18225. 24145401 10.1073/pnas.1307336110 PMC3831436

[33] Morwood SR, Nicholson LB. Modulation of the immune response by extracellular matrix proteins. Arch Immunol Ther Exp (Warsz). 2016;54:367–374. 10.1007/s00005-006-0043-x 17122884

[34] Black KE, Collins SL, Hagan RS, et al. Hyaluronan fragments induce IFNβ via a novel TLR4-TRIF-TBK1-IRF3-dependent pathway. J Inflam. 2013;10:1–9. 10.1186/1476-9255-10-23 PMC3682892 23721397

[35] Collins SL, Black KE, Chan-Li Y, et al. Hyaluronan fragments promote inflammation by down-regulating the anti-inflammatory A2a receptor. Am J Respir Cell Mol Biol. 2011;45:675–683. 21257926 10.1165/rcmb.2010-0387OC PMC3208614

[36] Marchant DJ, Bellac CL, Moraes TJ, et al. A new transcriptional role for matrix metalloproteinase-12 in antiviral immunity. Nat Med. 2014;20:493–502. 24784232 10.1038/nm.3508

[37] Soria-Valles C, Gutiérrez-Fernández A, Osorio FG, et al. MMP-25 Metalloprotease Regulates Innate Immune Response through NF-κB Signaling. J Iimmunol. 2016;197:296–302. 10.4049/jimmunol.1600094 27259858

[38] Orian-Rousseau V, Sleeman J. CD44 is a multidomain signaling platform that integrates extracellular matrix cues with growth factor and cytokine signals. Adv Cancer Res. 2014;123:231–254. 25081532 10.1016/B978-0-12-800092-2.00009-5

[39] Heldin P, Kolliopoulos C, Lin CY, et al. Involvement of hyaluronan and CD44 in cancer and viral infections. Cell Signal. 2020;65:109427. 31654718 10.1016/j.cellsig.2019.109427

[40] Murakami T, Kim J, Li Y, et al. Secondary lymphoid organ fibroblastic reticular cells mediate trans-infection of HIV-1 via CD44-hyaluronan interactions. Nat Commun. 2018;9:1–14. 29934525 10.1038/s41467-018-04846-w PMC6015004

[41] Iqbal J, Sarkar-Dutta M, McRae S, et al. Osteopontin regulates hepatitis C virus (HCV) replication and assembly by interacting with hcv proteins and lipid droplets and by binding to receptors αVβ3 and CD44. J Virol. 2018;92:e02116–17. 29669827 10.1128/JVI.02116-17 PMC6002707

[42] Puré E, Cuff CA. A crucial role for CD44 in inflammation. Trends Mol Med. 2001;7:213–221. 11325633 10.1016/s1471-4914(01)01963-3

[43] Wang X, Mbondji-Wonje C, Zhao J, et al. IL-1β and IL-18 inhibition of HIV-1 replication in Jurkat cells and PBMCs. Biochem Biophys Res Commun. 2016;473:926–930. 27049306 10.1016/j.bbrc.2016.03.153

[44] Xue M, Zhao J, Ying L, et al. IL-22 suppresses the infection of porcine enteric coronaviruses and rotavirus by activating STAT3 signal pathway. Antiviral Res. 2017;142:68–75. 28322925 10.1016/j.antiviral.2017.03.006 PMC7113769

[45] Bouezzedine F, Fardel O, Gripon P. Interleukin 6 inhibits HBV entry through NTCP down regulation. Virology. 2015;481:34–42. 25765005 10.1016/j.virol.2015.02.026

[46] Kuo TM, Hu CP, Chen YL, et al. HBV replication is significantly reduced by IL-6. J Biomed Sci. 2009;16:41. 19374779 10.1186/1423-0127-16-41 PMC2687430

[47] Nimmerjahn F, Dudziak D, Dirmeier U, et al. Active NF-κB signalling is a prerequisite for influenza virus infection. J Gen Virol. 2004;85:2347–2356. 15269376 10.1099/vir.0.79958-0

[48] Haas F, Yamauchi K, Murat M, et al. Activation of NF-κB via endosomal Toll-like receptor 7 (TLR7) or TLR9 suppresses murine herpesvirus 68 reactivation. J Virol. 2014;88:10002–10012. 24942583 10.1128/JVI.01486-14 PMC4136310

[49] Ding Z, An K, Xie LL, et al. Transmissible gastroenteritis virus infection induces NF-κB activation through RLR-mediated signaling. Virology. 2017;507:170–178. 28448848 10.1016/j.virol.2017.04.024 PMC7111708

[50] Cao L, Gao Y, Ren X, et al. Porcine epidemic diarrhea virus infection induces NF-κB activation through the TLR2, TLR3 and TLR9 pathways in porcine intestinal epithelial cells. J Gen Virol. 2015;96:1757–1767. 25814121 10.1099/vir.0.000133

[51] Wang Y, Sun A, Sun Y, et al. Porcine transmissible gastroenteritis virus inhibits NF-κB activity via nonstructural protein 3 to evade host immune system. Virol J. 2019;16:1–13. 31382996 10.1186/s12985-019-1206-9 PMC6683377

[52] Yang CH, Li HC, Ku TS, et al. Hepatitis C virus down-regulates SERPINE1/PAI-1 expression to facilitate its replication. J Gen Virol. 2017;98:2274–2286. 28857040 10.1099/jgv.0.000901

