# Supplementary material for: Role of intestinal extracellular matrix-related signaling in porcine epidemic diarrhea virus infection
Source: Virulence. 2021 Sep 13;12(1):2352–65. doi: 10.1080/21505594.2021.1972202 (PMC8451458; doi:10.1080/21505594.2021.1972202)
Supplement: Supplemental Material [file KVIR_A_1972202_SM8364.docx]

**
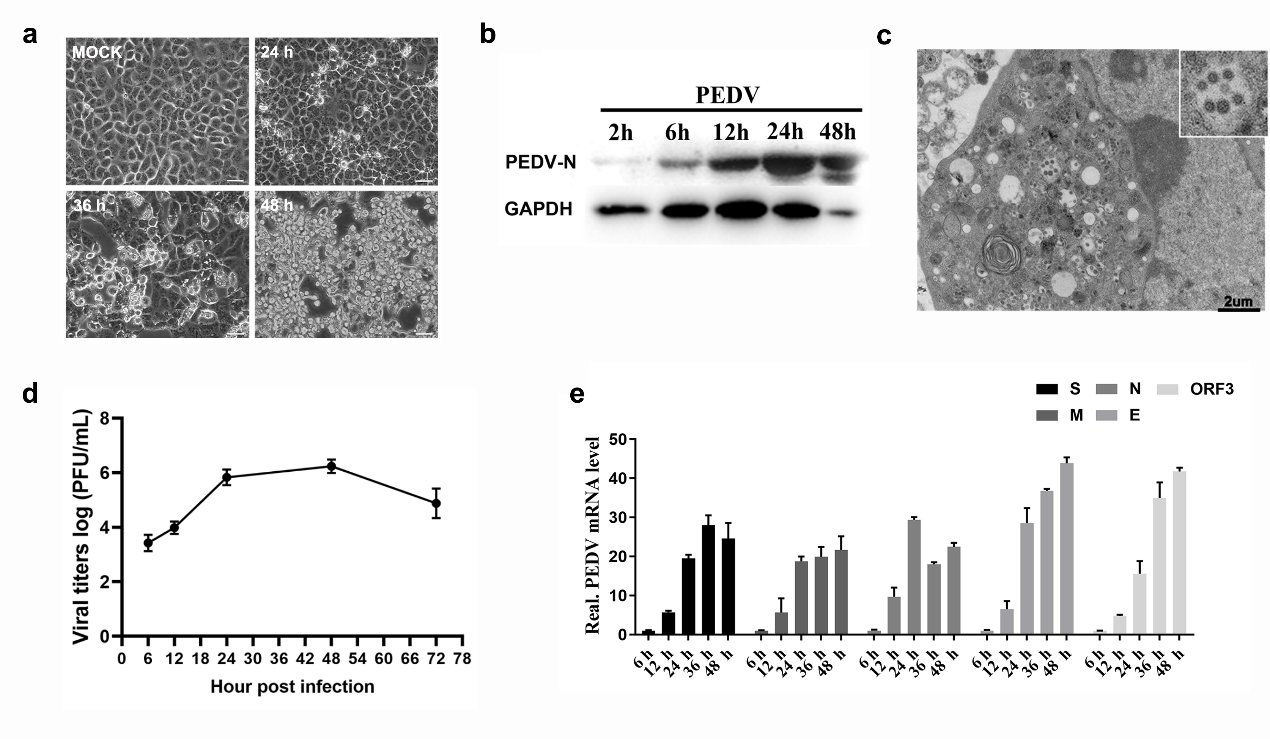
Figure S1. The propagation of PEDV in Vero cell.** (a) The cytopathic effects (CPE) exerted by Vero E6 cell under 12, 24 and 48 h when the infection of PEDV was achieved, and cell under the infection of mock under 48 h to be one control. (b) Western blot analysis of the N protein expression within Vero cell under the suggested time point after the infection of PEDV. GAPDH expressing state acted as one protein loading control. (c) TEM observation of Vero E6 cell under the infection of PEDV (MOI = 0.1), Scale bar, 2μm. The intracellular PEDV virions were displayed in higher-magnification views. (d) This study obtained the viral titer in Vero cells based on plaque assays at the indicated time points post the infection of PEDV. (e) Viral RNA level within Vero E6 cell were determined through detecting PEDV S gene, N gene, ORF3 gene, M gene and E gene at different time after PEDV infection.

**Figure S2. Differentially expression gene in PEDV-infected Vero E6 cells.**


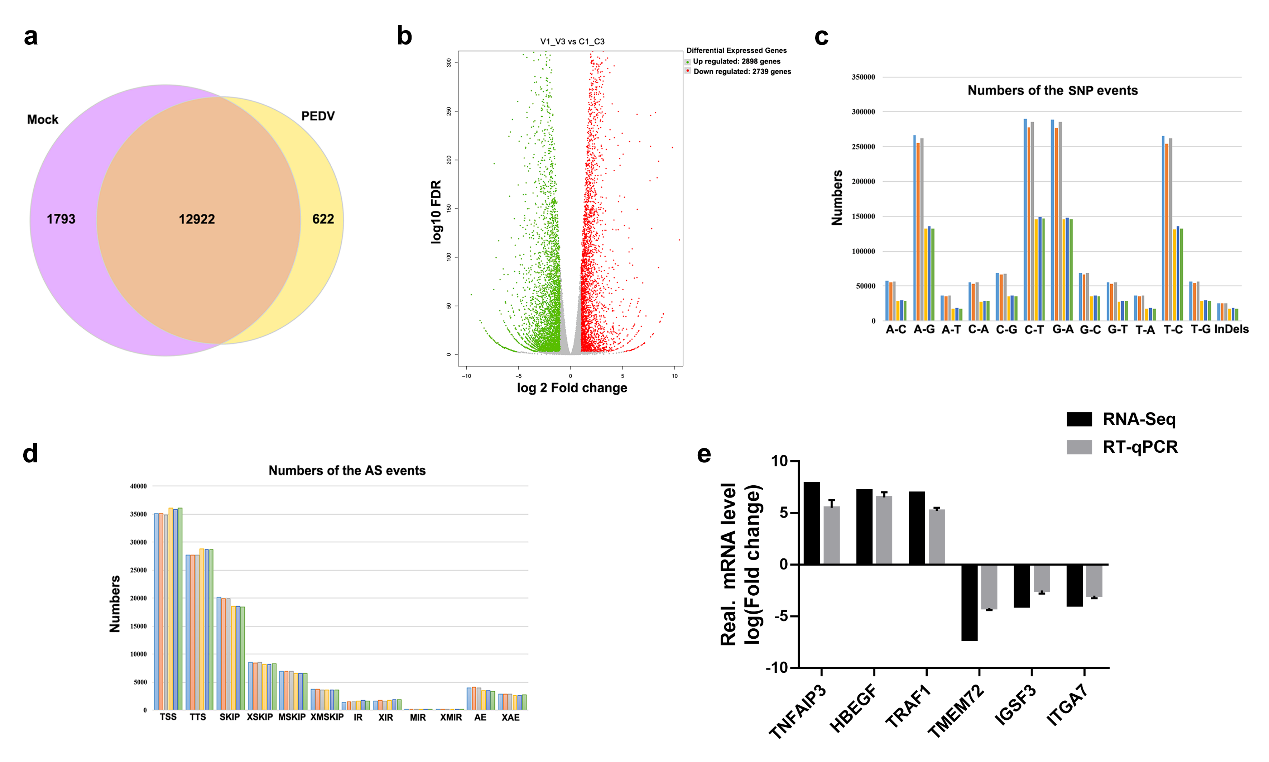

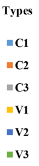

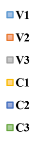


(a) Venn diagram presenting DEG overlap under the clustering process to PEDV vs Control represented by two ellipses. The number in the diagram indicated gene number refers to each comparison. (b) This study developed a volcano plot for visualizing the number of gene with the significant differential expression. The horizontal line indicates 0.05 FDR p-value, and the vertical line represents the two-fold variation within the expression log. The green or red dots represent gene with significant increase or decrease according to viral infection, respectively. (c and d) Statistical information for SNPs and AS event under the identiﬁcation according to RNA-Seq data. The x-axis indicates the type of SNP and AS event. The y-axis represents event number. (e) Randomly select differentially expressed genes for verification via RT-qPCR.


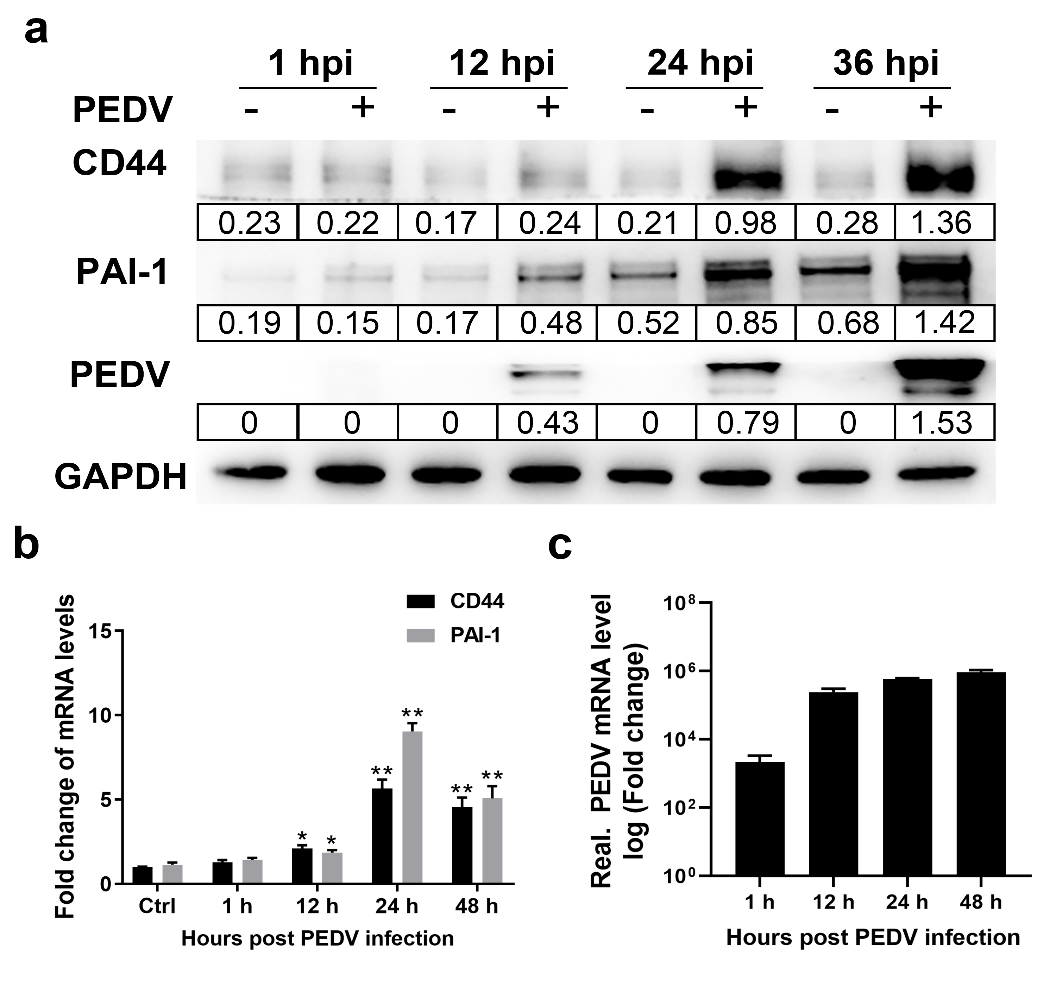


**Figure S3. PEDV infection enhance CD44 and PAI-1 expression in intestinal epithelial cells (IECs).** The intestinal epithelial cells (IECs) were isolated from one-week-old piglets. After reaching confluence, the IECs were infected with PEDV for indicated time (1-48 h), then cellular total proteins and RNA were harvested. (a) The protein expression of CD44, PAI-1 and PEDV-N was detected by western blotting. (b) Viral RNA levels in IECs were determined by detection of the PEDV N genes at different time after PEDV RT-qPCR. The kinetics of PEDV propagation in IECs was determined by detection of the PEDV N genes at different time after PEDV infection. The data represent means ± SDs of triplicate assays. * *P* < 0.05; ** *P* < 0.01.

**
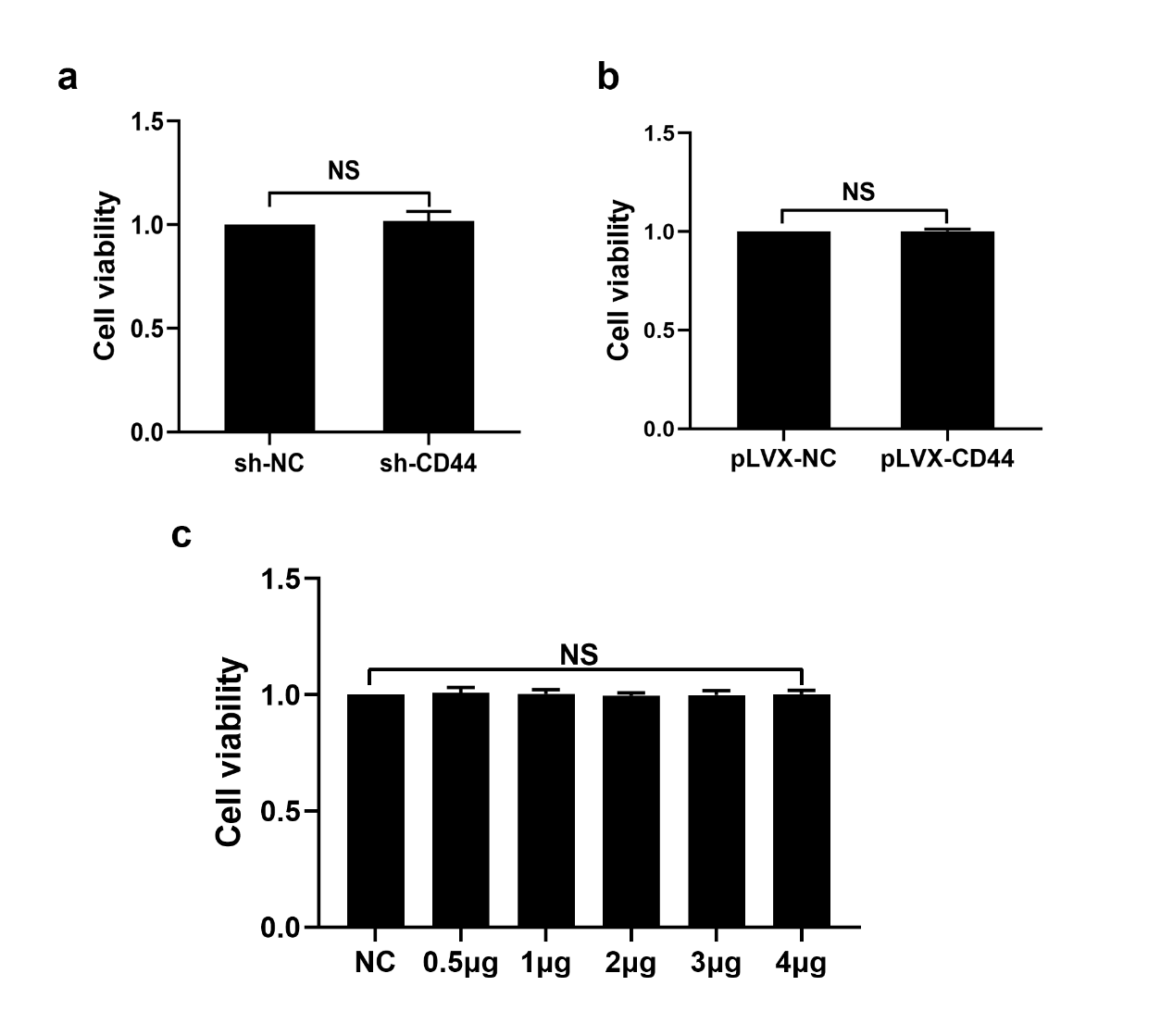
**

**Figure S4. CD44 knockdown and overexpression does not impact cell viability.** (a) Vero E6 cells stably expressing shRNA-NC or shRNA-CD44 under the plating and culturing process into seventy percent confluent monolayer in terms of CCK-8 test. (b) Vero E6 cells stably expressing pLVX-NC or pLVX-CD44 under the plating and culturing process into seventy percent confluent monolayer in terms of CCK-8 test. (c) After the 72-hour transient transfection of pLVX-CD44 with a different plasmid concentration, the cell viability pertaining to Vero E6 cell were detected based on CCK-8 assay.


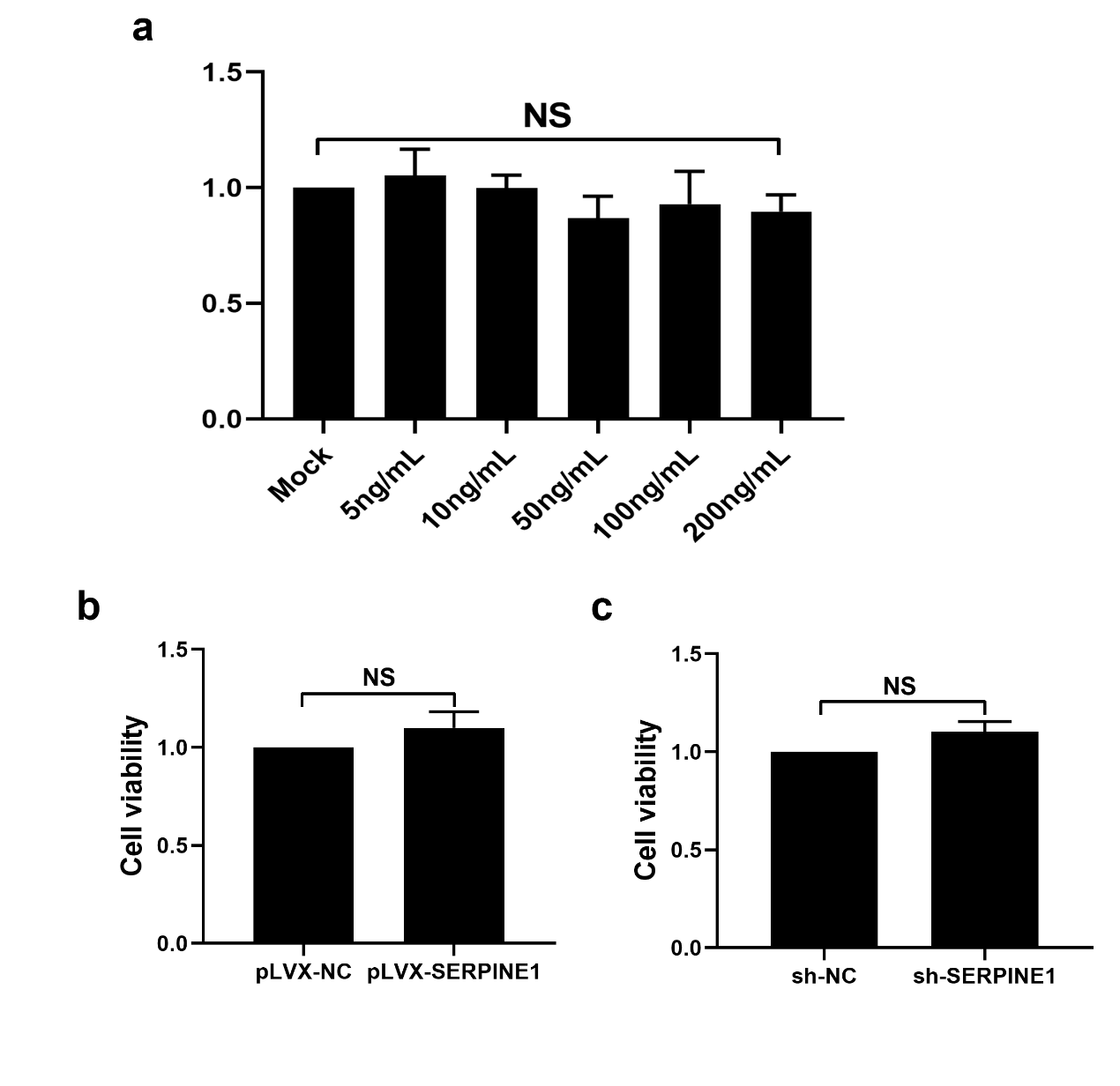


**Figure S5. PAI-1 treatment, knockdown and overexpression does not impact viability of cell.** (a) Viability of cell under the determination based on CCK-8 test when the Vero E6 cell received the 24-hour treatment by using PAI-1 under a range of concentrations. (b) Vero E6 cells stably expressing shRNA-NC or shRNA-SERPINE1 under the plating and culturing process into seventy percent confluent monolayer in terms of CCK-8 test. (c) Vero E6 cells stably expressing pLVX-NC or pLVX-SERPINE1 under the plating and culturing process into seventy percent confluent monolayer in terms of CCK-8 test.

**
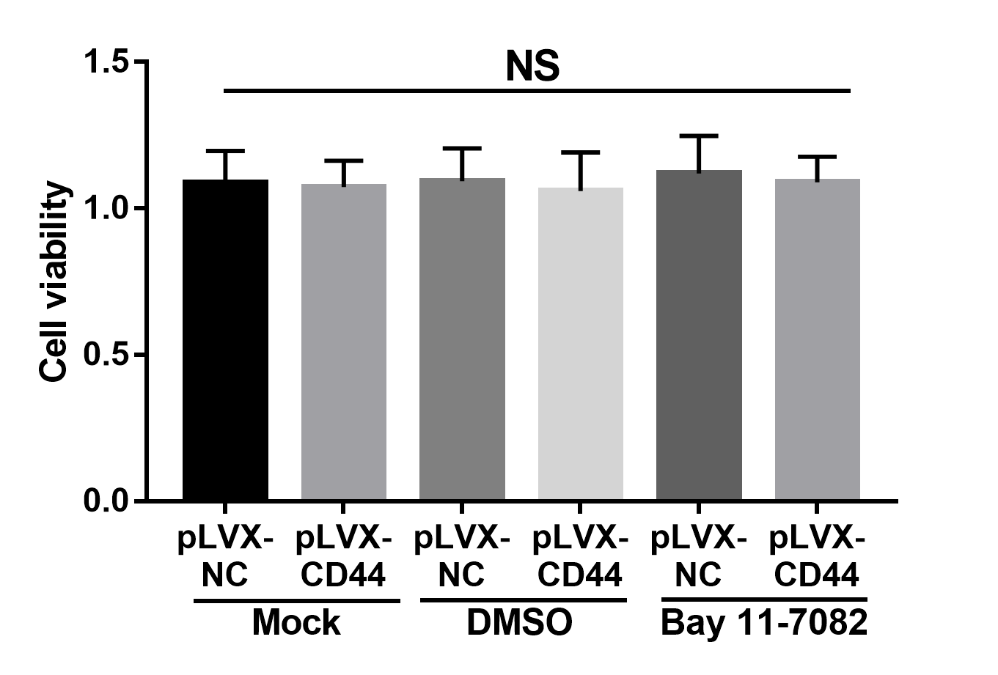
**

**Figure S6. BAY 11-7082 treatment does not impact viability of cell.** Viability of cell under the determination based on CCK-8 test when cells received the 2-hour treatment by using 10 μM Bay 11-7082 or DMSO.

**Table S1.** **Primer sequences used for plasmid construction**

| **Genes** | **Primers** | **Sequence (5'-3')** |
| --- | --- | --- |
| shRNA  CD44-1 | Forward | GATCCGGAGGTTACAUCTTTCACATTCAAGAGATGTGAAAGATGTAACCTCCTTTTTG |
|  | Reverse | AATTCAAAAAGGAGGTTACATCTTTCACATCTCTTGAATGTGAAAGATGTAACCTCCG |
| shRNA  CD44-2 | Forward | GATCCGCAGGTACGGGTTCATAGATTCAAGAGATCTATGAACCCGTACCTGCTTTTTG |
|  | Reverse | AATTCAAAAAGCAGGTACGGGTTCATAGATCTCTTGAATCTATGAACCCGTACCTGCG |
| shRNA  CD44-3 | Forward | GATCCGAAAGCCAAGTGGACTCAATTCAAGAGATTGAGTCCACTTGGCTTTCTTTTTG |
|  | Reverse | AATTCAAAAAGAAAGCCAAGTGGACTCAATCTCTTGAATTGAGTCCACTTGGCTTTCG |
| shRNA Serpine1-1 | Forward | GATCCGCTTCATGCCCCACTTCTTTTCAAGAGAAAGAAGTGGGGCATGAAGCTTTTTG |
|  | Reverse | AATTCAAAAAGCTTCATGCCCCACTTCTTTCTCTTGAAAAGAAGTGGGGCATGAAGCG |
| shRNA Serpine1-2 | Forward | GATCCGCCCTCACCAACATTCUGATTCAAGAGATCAGAATGTTGGTGAGGGCTTTTTG |
|  | Reverse | AATTCAAAAAGCCCTCACCAACATTCTGATCTCTTGAATCAGAATGTTGGTGAGGGCG |
| shRNA Serpine1-3 | Forward | GATCCGGCCAAGTGATGGAACCTTTTCAAGAGAAAGGTTCCATCACTTGGCCTTTTTG |
|  | Reverse | AATTCAAAAAGGCCAAGTGATGGAACCTTTCTCTTGAAAAGGTTCCATCACTTGGCCG |

**Table S2.** **Primers used for real-time PCR**

| **Genes** | **Primers** | **Sequence (5'-3')*** |
| --- | --- | --- |
| PEDV (N) | Forward | CACCTCCTGCTTCACGTACA |
|  | Reverse | AGCTCCACGACCCTGGTTAT |
| GAPDH  (Sus scrofa) | Forward | TCATCATCTCTGCCCCTTCT |
|  | Reverse | GTCATGAGTCCCTCCACGAT |
| GAPDH  (Chlorocebus sabaeus) | Forward | ACATCATCCCTGCCTCTACTG |
|  | Reverse | CCTGCTTCACCACCTTCTTG |
| CD44  (Sus scrofa) | Forward | CACCCAGACCAAGAAGGTCC |
|  | Reverse | TGGCATGTGAGCTGCTGTAT |
| CD44  (Chlorocebus sabaeus) | Forward | TGTTAACCCCGATGGCACTC |
|  | Reverse | TGCATCTTGGTCTCTGGTAGC |
| SERPINE1  (Sus scrofa) | Forward | CTAACCAGGCGGACTTCTCA |
|  | Reverse | AATGGCCGTAGAGGAGGATG |
| SERPINE1  (Chlorocebus sabaeus) | Forward | AGAGGGTAGGGCACAAAGAC |
|  | Reverse | CAGGGCTCTTGGTATGTTGC |
| IL-6  (Chlorocebus sabaeus） | Forward | TGTGAAAGCAGCAAAGAG |
|  | Reverse | AGTGTCCTCATTGAATCCA |
| IL-11  (Chlorocebus sabaeus） | Forward | CCCGAGTGTGCTGACAAGG |
|  | Reverse | CCTGAAGACCCTGGAGCCTG |
| IL-18  (Chlorocebus sabaeus） | Forward | GAGCCTATTTGAAGGCGGGA |
|  | Reverse | CAGAGTTGGCAGCTAGGAGG |
| DB-1  (Chlorocebus sabaeus） | Forward | ATAAGCCGCGGAGACATCAG |
|  | Reverse | CTCCTCAGAAAGTGAGCCCC |
